# Intussusception through an ileostomy: A case report and literature review

**DOI:** 10.1016/j.amsu.2019.09.003

**Published:** 2019-09-18

**Authors:** M. Maatouk, Y. Ben Safta, Aymen Mabrouk, Marwa Bouafif, Nesrine Hajdahmen, Anis Ben Dhaou, Sami Daldoul, Sofien Sayari, Karim Haouet, Mounir Ben Moussa

**Affiliations:** University Tunis El Manar, Faculty of Medicine of Tunis, Surgery A Department, Charles Nicolle Hospital, France

**Keywords:** Intussusception, Ileostomy, Surgical emergency

## Abstract

**Introduction:**

Intussusception through an ileostomy is one of the rarest complications of stomas. In this study we report a case and a brief update of the literature to focus on the clinical level of this pathology and the therapeutic attitudes.

**Presentation of case:**

a 44-year-old man who underwent a small bowel resection with double stoma for tuberculosis peritonitis presented with stomatal prolapse. On examination of the stoma, small bowel mucosa appeared to have evident rather than serosa. The patient had an elective reduction of the proximal stoma under anesthesia.

**Conclusion:**

A review of the literature shows that Intussusception through an ileostomy can occur at any time after the first surgery. The cause is still unclear. Urgent conservative surgical management based on manual reduction should be preferred.

## Highlights

• Intussusception through an ileostomy is one of the rarest complications of stomas.
• The intussusception through an ileostomy is a surgical emergency because of the risk of intestinal necrosis.
• The present case of Intussusception through an ileostomy is treated by an elective reduction of the proximal stoma..
• In most of cases, there is at least one intra-abdominal pressure-increasing factor.

## 1 Introduction

Stoma has become a common surgical act, since described by Brook in 1952 [1].It is still used in several surgeries. Stoma can be responsible for 35% of patient's postoperative complications [2]. The most frequent complications are the ileostomy retraction (40%), the avulsion (24%), stomatal necrosis (9%) and prolapse (2%) [3]. The intussusception through an ileostomy has been rarely described in the medical literature. The intussusception is the invagination of the intestine through a loop, or less frequently an end ileostomy following the normal flow of peristalsis or rarely, against it [4]. This complication may result in an intestinal necrosis because of the compression of the blood vessels. Gangrene is the most feared complication. In this study, we report a case and a brief update of the literature to focus on the interest of this pathology on the clinical level and therapeutic attitudes.

## 2 Clinical presentation

This work has been reported in accordance with the SCARE criteria [5]. The case is about a 44-year-old man with body mass index of 22 kg/m2. His past medical history included post-viral hepatitis C and pulmonary tuberculosis treated and there was no other systemic illness. Patient had a 50 pack-year history of smoking. His family history was not relevant.

Seven months ago, he underwent an operation for tuberculosis peritonitis for which he had a small bowel resection with double end ileostomy. The ileostomies were fixed with a series of sutures (2/0 vicryl; ethicon). The sutures were placed through the dermis, followed by a seromuscular bite, away from the mesentery, from the proximal to the distal end of the ileum. He is started on isoniazid 300 mg/day, pyrazinamide 2 g/day and streptomycin 1 g intramuscularly after the operation. The antituberculous therapy ceased at the end of the sixth month.

He was in clinical remission and stopped treatment. He consulted for the appearance of stomatal prolapse that was irreducible since 6 h, with no transit disorder or other associated signs. During the clinical examination, the patient was conscious with stable hemodynamic constants and a flexible depressible abdomen.

**Fig. 1:**
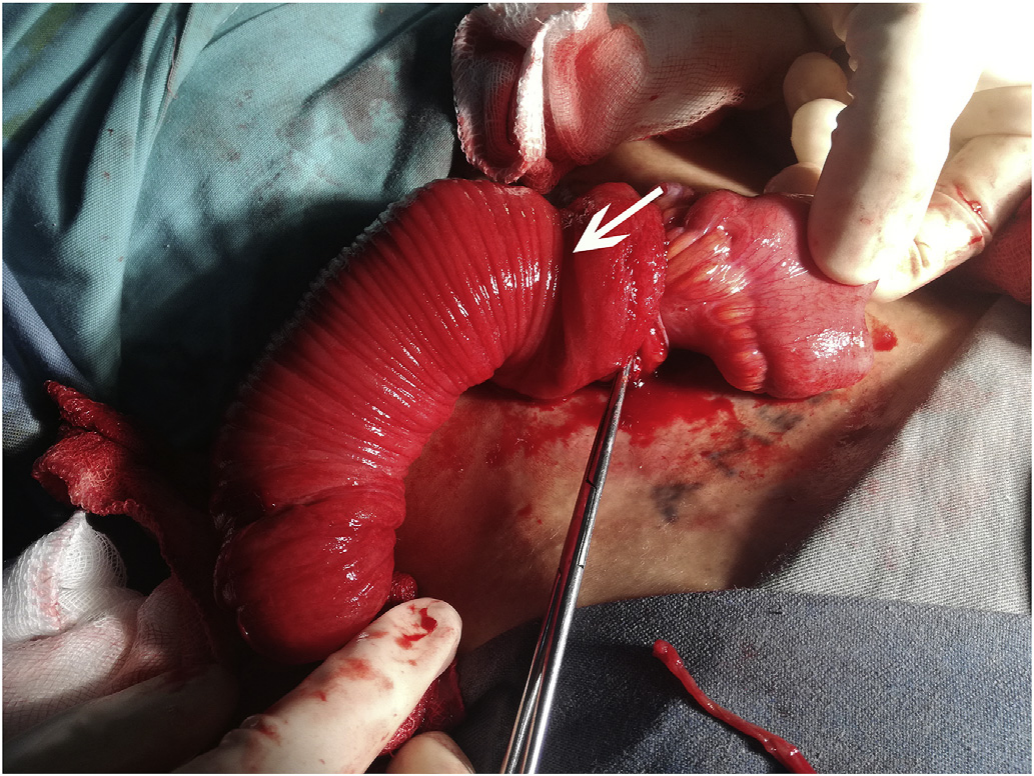
Intraoperative aspect of intussusception through ileostomy.

On examination of the stoma the proximal limb lumen of the ileostomy was normal. Nevertheless there was 10 cm of small bowel protruding through the site of the distal limb. Small bowel mucosa was evident rather than serosa. The diagnosis of retrograde intussusception was made. The proximal limb of the ileostomy was irreducible edematous (Fig. 1).

As there aren't signs of necrosis, the patient had an elective reduction of the proximal stoma under anesthesia.

The intussusception was attempted manually by applying gentle pressure with no bowel damage.

The patient recovered well and was discharged from the hospital on the first postoperative day with excellent ileostomy function.

## 3 Discussion

### 3.1 Etiology and physiopathology

Contrasting with the commonly observed adult intussusception, which is secondary to an organic cause, the one through ileostomy is due to a probable rise of intra-abdominal pressure [4]. The first three cases described in literature were related to pregnant women. According to this, authors supposed that the pregnancy causes the intussusception by increasing the intra-abdominal pressure.

Cases recently published discriminate against the presence of a pressure rise factor.

One resemblance between our case and the other reported cases is the miss of a known lead point as an etiology for the intussusception. Therefore, the mechanism of the intussusception through the ileostomy is still unknown [6].

### 3.2 Literature review

Bibliography research is made with Pubmed using the key words “intussusception AND Ileostomy”. The research result was 47 articles. We included in this literature review all case reports found. Nine studies were published from 1959 to 2018; the clinical observations are summarized in the (Table 1). Lateral or terminal intussusception of the ileostomy can actually occur at any time postoperatively. The reported cases were observed starting the 3rd postoperative day and up to ten years after performing the stoma. In 60% of the cases, there is at least one intra-abdominal pressure-increasing factor like: pregnancy, ascites, uncontrollable vomiting and chronic cough. The surgeon must determine the risky subjects to avoid the intussusception of the stoma complication [7].

**Table 1:** Cases reported in the literature of invaginations through an ileostomy.

	Age/Gender	Etiology	Cause of the ileostomy	Delay∗	Type of ileostomy	Necrosis	Treatment
**Priest 1959** [10]	52/F	Pregnancy	Acute colitis	10 years	terminal	Necrosis	Reduction + resection by elective
**Adedeji 1992** [11]	31/F	Pregnancy	Acute colitis	8 years	terminal	No necrosis	Failure of manual reduction ➔laparotomy
**Kwok 2005** [4]	32/F	Pregnancy	Cohn's diseas e	–	terminal	No necrosis	Failure of manual reduction ➔laparotomy
**Chessin 2006** [13]	49/M	ascites	Complicated diverticulitis	16 weeks	lateral	Necrosis	Reduction + resection by elective
**Chen 2009** [9]	91/F	–	Rectal perforation	1 month	lateral	No necrosis	Failure of manual reduction ➔laparotomy
**Bielecki 2010** [12]	36/F	–	Cohn's disease	6 days	terminal	No necrosis	laparotomy
**Khan 2011** [8]	72/M	Vomiting	Rectal cancer	3 days	lateral	No necrosis	Failure of manual reduction ➔laparotomy
**Bhange 2013** [7]	72/M	Cough	Sigmoid cancer	4 weeks	terminal	No necrosis	laparotomy
**Jung 2017** [6]	49/M	–	Ogilvie syndr ome	4 months	lateral	Necrosis	Reduction + resection by elective
**Our case 2019**	44/M	–	Tuberculin peritonitis	7 months	lateral	No necrosis	Elective manual reduction

∗ Delay between ileostomy and intussusception.

### 3.3 Diagnosis and therapeutic management

The diagnosis of the Intussusception through ileostomy is based on clinical examination. The only diagnosis difficulty reported is related to the strangulated stomatal prolapse. In fact, the intussusception is commonly characterized by the replacement of the outermost layer “the serous” by the mucous membrane with its conniving valves [8]. Surgery remains the only possible treatment. The use of radiological explorations is not necessarily required for the diagnosis of the intussusception through ileostomy. Ran Jung reported the only case of intussusception through an ileostomy investigated by an abdominal CT scan in order to detect abdomen intestinal necrosis [6].The intussusception through an ileostomy is a surgical emergency because of the risk of intestinal necrosis. The treatment must be conservative. It is classically based on a manual reduction under general anesthesia with probability of conversion to laparotomy in case of failure of the non-invasive reduction [9]. Interestingly, such our case and according to the literature half of patients had a conservative treatment with an elective reduction. Thus, there is no resort to a midline incision in the absence of intestinal necrosis or unsuccess of the manual reduction.

## 4 Conclusion

Intussusception through an ileostomy is one of the rarest complications of stomas. It should be rapidly treated to avoid risks of intestinal necrosis. It requires more conservative urgent surgical intervention based on manual reduction.

## Consent

Written informed consent was obtained from the patient for publication of this case.

## Provenance and peer review

Not commissioned, externally peer reviewed.

## Ethical approval

We have reported a single case with no requirement for ethical approval. This manuscript does not describe a clinical study. Written informed consent was obtained from the patient for publication of this case.

## Sources of funding

No source of funding.

## Author contribution

Maatouk M: study concept, Data collection, data analysis or interpretation, writing the paper.

Ben Safta Y: study concept, writing the paper.

Mabrouk A: study concept, writing the paper.

Bouafif M: data collection.

Hadj Dahmane N: data collection, data analysis or interpretation.

Ben Dhaou A: data collection, data analysis or interpretation.

Daldoul S: data collection, data analysis or interpretation.

Sayari S: data collection, data analysis or interpretation.

Haouet K: advised and designed the report.

Ben Moussa M: advised and designed the report.

## Conflicts of interest

No potential conflict of interest relevant to this article was reported.

## Research registration number

N/A.

## Guarantor

Dr Yacine Ben Safta.
